# Can we predict neurological complications in patients with metastatic spinal tumors?

**DOI:** 10.3389/fonc.2025.1625545

**Published:** 2025-08-19

**Authors:** Chul Jung, Hee Tae Shin, Cho Rong Bae, Joon Hee Lee, Jin Hoon Park, Jae-Hwan Cho, Choong Guen Chee, Jae Yong Jeon

**Affiliations:** ^1^ Department of Rehabilitation Medicine, Asan Medical Center, University of Ulsan College of Medicine, Seoul, Republic of Korea; ^2^ Department of Neurosurgery, Asan Medical Center, University of Ulsan College of Medicine, Seoul, Republic of Korea; ^3^ Department of Orthopedic Surgery, Asan Medical Center, University of Ulsan College of Medicine, Seoul, Republic of Korea; ^4^ Department of Radiology, Seoul National University Bundang Hospital, Seongnam, Republic of Korea

**Keywords:** metastatic spinal tumor, spinal cord injury, neurological complication, epidural spinal cord compression scale, spinal instability neoplastic score, radicular pain

## Abstract

**Background:**

Previous studies primarily analyzed spinal cord injuries in patients with metastatic spinal tumors after such injuries had already occurred. This study aimed to determine whether clinical and radiological factors are associated with the occurrence and severity of newly developed spinal cord injuries within 1 year in patients with metastatic spinal tumors.

**Methods:**

We retrospectively examined patients with metastatic spinal tumors who were referred to the Department of Rehabilitation Medicine between 2017 and 2021. Using patients’ clinical data and magnetic resonance imaging (MRI) findings, we investigated whether pain characteristics, Spinal Instability Neoplastic Score (SINS), and Epidural Spinal Cord Compression (ESCC) grades were associated with the occurrence and severity of spinal cord injuries within 1 year of MRI evaluation.

**Results:**

Among the 70 included patients, 40 developed spinal cord injuries. Multivariate analysis identified an ESCC grade 2 or 3 (high-grade ESCC) as the only significant predictor of spinal cord injury within 1 year (P = 0.016). Higher ESCC grades were also significantly associated with a shorter time to onset of spinal cord injuries (P = 0.003). Regarding the severity of spinal cord injuries, the total score and categories of SINS were significantly higher in the mild deficit group than in the moderate to severe deficit group (P = 0.024 and P = 0.049, respectively).

**Conclusions:**

In patients with metastatic spinal tumors, high-grade ESCC was a significant predictor of spinal cord injury within 1 year and was associated with an earlier onset of spinal cord injury. Radicular pain and unstable spines based on SINS categories were also strongly associated with the occurrence of spinal cord injury. This study provides valuable insights for predicting 1-year functional outcomes and determining management strategies for spinal metastases.

## 1 Introduction

In patients with advanced-stage cancers, metastatic spinal tumors are occasionally encountered (1). Advances in treatment modalities, such as stereotactic radiosurgery and minimally invasive surgery, have shifted research interest from survival rates to quality of life in patients with metastatic spinal tumors (2, 3). Spinal cord compression occurs in 5%–14% of patients with metastatic spinal tumors, often resulting in neurological complications, particularly spinal cord injury (1). These injuries can decrease both patients’ quality of life and overall survival (4, 5), highlighting the importance of appropriate treatment selection (6). Recently, the NOMPRS framework, which incorporates six key factors (neurologic, oncologic, medical, pain, rehabilitation, and support status), has been proposed to guide clinical decision-making (7).

Surgical intervention is commonly indicated in cases of metastatic spinal tumors with spinal instability or spinal cord compression resulting in spinal cord injuries (8, 9). The Spinal Instability Neoplastic Score (SINS) is a standard tool for evaluating spinal instability and guiding treatment (10, 11). Studies have demonstrated favorable responses to palliative radiotherapy in patients with metastatic spinal tumors having low-grade SINS, whereas those with high-grade SINS exhibited a significantly higher rate of radiotherapy failure (12). The degree of spinal cord compression can be graded using the Epidural Spinal Cord Compression (ESCC) scale, which evaluates cerebrospinal fluid space impingement and spinal cord compression based on magnetic resonance imaging (MRI) findings (13).

Both scoring systems have proven to be reliable tools for assessing spinal instability and spinal cord compression. Although pain characterization is integrated in the SINS scoring system, both systems have limitations, particularly in assessing neurologic deficits—an essential factor for surgical decision-making. Few studies have investigated the relationship between spinal cord injuries and both scoring systems in patients with metastatic spinal tumors (14–17). However, these analyses were primarily conducted after the occurrence of spinal cord injuries. This limits the clinical utility of these scoring systems, especially in patients with spinal metastases who have not yet developed neurological symptoms.

Beyond radiologic evaluation of spinal instability and spinal cord compression, 83%–95% of patients with metastatic spinal tumors clinically experience pain (18). This pain arises from factors, including vertebral body fracture, periosteal pressure, and nerve root compression (18–20). Identifying pain as a predictor of spinal cord injury may enhance clinical decision-making.

This study aimed to determine whether the SINS, ESCC grade, and clinical factors are associated with the occurrence and severity of spinal cord injuries within 1 year in patients with metastatic spinal tumors who are neurologically asymptomatic.

## 2 Materials and methods

### 2.1 Study design and population

In this single-center retrospective cohort study, we reviewed the medical records of patients with metastatic spinal tumors who were referred to the Department of Rehabilitation Medicine between January 2017 and May 2021. Patients aged ≥ 18 years, with radiologically confirmed spinal metastasis, regardless of the primary cancer type, were considered eligible for the study. Only patients with available whole-spine MRI data obtained within 1 year prior to referral were included. Patients were excluded if they had brain metastases, underwent whole-spine MRI after the occurrence of a spinal cord injury, or if ESCC grades could not be determined (e.g., when the level of spinal metastasis was below the level of the conus medullaris or when no axial images were available from whole-spine MRI).

After applying the exclusion criteria, the remaining patients were classified into two groups based on whether they developed spinal cord injuries within 1 year post-MRI. Clinical and radiologic data were then compared between the two groups. The study protocol was reviewed and approved by our institution’s ethics committee (IRB No. 2021-0373).

### 2.2 Data collection

Data were collected from electronic medical records. The clinical data included age, sex, type of primary cancer, type of treatments for spinal metastases (including those administered either within 1 year post-MRI or between the MRI and onset of spinal cord injury), pain characteristics, as well as the occurrence and severity of spinal cord injuries. The severity of spinal cord injury was assessed using the American Spinal Injury Association Impairment Scale (AIS) (21). The radiologic data analyzed in this study included the SINS (**Supplementary Table 1**) and ESCC grades (**Supplementary Table 2**).

#### 2.2.1 Pain characteristics and severity of spinal cord injuries

Pain characteristics in patients with metastatic spinal tumors were classified into three classical categories (18–20, 22). Localized back pain, typically described as deep and aching, is associated with tumor growth that causes periosteal stretching and inflammation. Mechanical back pain arises from increased strain on the muscles, tendons, ligaments, and joint capsules, often due to spinal deformity or collapse. This type of pain suggests impending or established spinal instability and is typically exacerbated by movement. Radicular pain is characterized by a lancinating quality of pain traveling into the limb along a specific dermatome. This indicates compression of nerve roots by tumors or pathologic fractures as the roots exit the spine.

The severity of spinal cord injuries was evaluated using the AIS. This scale consists of four grades: AIS A to AIS D (21). Patients classified as AIS D, exhibiting a considerable degree of muscle strength preservation below the neurological level, are considered to be ambulatory (14, 15). For the purpose of this study, patients with spinal cord injury were further categorized into two groups: mild deficit (AIS D) and moderate to severe deficit (AIS C, B, and A).

#### 2.2.2 SINS and ESCC scale

Spinal stability was evaluated using the SINS, which comprises six elements encompassing pain characteristics and radiologic findings. Patients were classified into three categories based on their total scores: stable (0–6), intermediate (7–12), and unstable spines (13–18) (8, 10). SINS was analyzed in two separate ways—one using the numerical scores themselves and the other using categorical variables.

The extent of epidural tumor invasion was evaluated using the ESCC scale, which is also known as the Bilsky scale (13). This scale assigns grades from 0 to 3 based on the degree of spinal cord compression due to the presence of tumors on the MRI. High-grade ESCC was defined as spinal cord deformation accompanied by partial (grade 2) or complete (grade 3) obliteration of the cerebrospinal fluid space. In patients with multiple spinal metastases, we evaluated the vertebral segment with the highest SINS or ESCC grades on MRI, or the segment ultimately responsible for spinal cord injury. All SINS and ESCC ratings were performed by a musculoskeletal radiologist with 8 years of experience (C.G.C).

### 2.3 Outcomes

The primary outcome was to identify predictors associated with the occurrence and severity of spinal cord injury within 1 year in patients with metastatic spinal tumors. As a secondary outcome, the association between SINS and ESCC grades and the interval from MRI to the onset of spinal cord injuries was investigated.

### 2.4 Statistical analysis

All statistical analyses were performed using PASW Statistics 18 (SPSS Inc., Chicago, IL). A P-value <0.05 was considered statistically significant. Continuous variables are presented as mean ± standard deviation and categorical variables as percentages. The Student’s t-test, Mann–Whitney U test, Pearson’s χ2 test, and Fisher’s exact test were used to compare variables between patients with and without spinal cord injuries, as well as between the groups with varying injury severities. Multivariate logistic regression analysis with stepwise variable selection was used to identify predictors of spinal cord injuries. Variables with a P-value <0.05 in the univariate analysis were considered as possible predictors and were subsequently included in the multivariate analysis. As a secondary analysis, the Jonckheere–Terpstra test was employed to analyze the ordered difference in the time to the onset of spinal cord injury according to the SINS and ESCC grades.

## 3 Results

### 3.1 Study population and baseline characteristics

A total of 296 patients with metastatic spinal tumors were initially eligible for this study. Of these, 226 patients were excluded for the following reasons: concomitant brain metastases (n = 59); absence of whole-spine MRI scans obtained within 1 year of referral and before spinal cord injury onset (n = 114); inability to perform ESCC scoring (n = 32); and insufficient data (n = 21). Consequently, 70 patients were included in the final analysis, of whom 40 (57.1%) developed a spinal cord injury within 1 year post-MRI and were classified into the spinal cord injury group.

**Table 1** summarizes the clinical characteristics of the included patients. Breast cancer was the most common primary cancer (n = 18, 25.7%), followed by lung, prostate, kidney, and liver cancers. Other cancers included urothelial cell carcinoma and multiple myeloma (n = 3 each) and 10 additional cancer types (n ≤ 2 each). Spinal metastases were primarily located in the thoracic spine. Combined systemic and radiation therapy was the most common treatment modality for spinal metastases (n = 30, 42.9%). Surgery was more frequently performed in the spinal cord injury group, whereas systemic therapy was more frequently administered in the no spinal cord injury group.

**Table 1:** Baseline characteristics of the study population.

	All patients (n = 70)	Spinal cord injury (n = 40)	No spinal cord injury (n = 30)
Patient demographics			
Male sex, n (%)	35 (50.0)	23 (57.5)	12 (40.0)
Age, years, mean (SD)	58.9 (11.7)	57.0 (11.0)	61.5 (12.3)
Tumor-related			
Site of primary cancer, n (%)			
Breast	18 (25.7)	7 (17.5)	11 (36.7)
Lung	15 (21.4)	7 (17.5)	8 (26.7)
Prostate	7 (10.0)	4 (10.0)	3 (10.0)
Kidney	6 (8.6)	5 (12.5)	1 (3.3)
Liver	5 (7.1)	2 (5.0)	3 (10.0)
Others	19 (27.1)	15 (37.5)	4 (13.3)
Level of spinal metastases, n (%)			
Cervical	7 (10.0)	3 (7.5)	4 (13.3)
Thoracic	53 (75.7)	35 (87.5)	18 (60.0)
Lumbar	10 (14.3)	2 (5.0)	8 (26.7)
Treatment-related			
Systemic therapy, n (%)	14 (20.0)	4 (10.0)	10 (33.3)
Radiation therapy, n (%)	15 (21.4)	10 (25.0)	5 (16.7)
Combined systemic and radiation therapy, n (%)	30 (42.9)	17 (42.5)	13 (43.3)
Surgery, n (%)	11 (15.7)	9 (22.5)	2 (6.7)

SD, standard deviation.

### 3.2 Occurrence of spinal cord injuries

The comparison of clinical and radiologic variables between groups with and without spinal cord injuries is presented in **Table 2**. All three pain categories were more prevalent in the spinal cord injury group on the day of the MRI; however, only radicular pain reached statistical significance (P = 0.017). The total SINS score did not differ significantly between the groups. In contrast, the distribution of SINS categories and ESCC grades differed significantly (P = 0.017 and 0.004, respectively). Notably, patients with higher ESCC grades exhibited a higher prevalence of spinal cord injuries. All patients classified with unstable SINS and ESCC grade 3 developed spinal cord injuries within 1 year.

**Table 2:** Comparison of characteristics between the patient groups with and without spinal cord injuries.

	Spinal cord injury (n = 40)	No spinal cord injury (n = 30)	P-value
Clinical			
Pain character, n (%)			
Localized pain	16 (40.0)	11 (36.7)	0.777
Mechanical pain	13 (32.5)	6 (20.0)	0.244
Radicular pain	19 (47.5)	6 (20.0)	0.017
Radiologic			
SINS, total score, mean (SD)	8.3 (3.6)	7.8 (2.3)	0.457
SINS, category, n (%)			
Stable	16 (40.0)	7 (23.3)	
Intermediate	19 (47.5)	23 (76.7)	
Unstable	5 (12.5)	0 (0.0)	0.017
ESCC scale, n (%)			
0	10 (25.0)	18 (60.0)	
1	12 (30.0)	8 (26.7)	
2	10 (25.0)	4 (13.3)	
3	8 (20.0)	0 (0.0)	0.004

The Student’s *t*-test, Pearson’s χ2 test, and Fisher’s exact test were used to compare variables between the patient groups.

SINS, spinal instability neoplastic score; SD, standard deviation; ESCC, epidural spinal cord compression.

The association between variables and the occurrence of spinal cord injury was assessed using an univariate logistic regression analysis (**Table 3**). Because all patients with unstable SINS categories and ESCC grade 3 were included in the spinal cord injury group, performing an analysis using the original SINS category and ESCC grade was not possible. Instead, converted binary variables (SINS ≥ 10 and high-grade ESCC) were included in the analysis. In a multivariate analysis, high-grade ESCC was identified as the only statistically significant predictor of spinal cord injury (odds ratio [OR], 4.653; 95% confidence interval [CI], 1.329–16.297). Although not statistically significant, radicular pain was associated with an increased risk of spinal cord injury (OR, 3.079; 95% CI, 0.985–9.621).

**Table 3:** Multivariate logistic regression analysis for the patient group with spinal cord injuries.

Variables	Univariate analysis		Multivariate analysis	
	OR (95% CI)	P-value	OR (95% CI)	P-value
Clinical				
Pain character				
Localized pain	1.152 (0.434, 3.054)	0.777		
Mechanical pain	1.926 (0.633, 5.860)	0.248		
Radicular pain	3.619 (1.218, 10.751)	0.021	3.079 (0.985, 9.621)	0.053
Radiologic				
SINS ≥ 10	1.769 (0.609, 5.141)	0.295		
High-grade ESCC ^a^	5.318 (1.565, 18.071)	0.007	4.653 (1.329, 16.297)	0.016

The odds of neurologic complications for each variable were analyzed using logistic regression. Variables with P-values <0.05 in the univariate analysis were included in the multivariate analysis. ^a^ High-grade ESCC was defined as ESCC grades of 2 and 3.

OR, odds ratio; CI, confidence interval; SINS, spinal instability neoplastic score; ESCC, epidural spinal cord compression.

### 3.3 Severity of spinal cord injuries

The spinal cord injury group was further subdivided into the following two groups based on the severity of spinal cord injuries: mild deficit (n = 22) and moderate to severe deficit (n = 18). **Table 4** presents a comparison of variables between these two groups. No statistically significant differences were observed in pain categories or the period between the MRI and the onset of spinal cord injury. However, the mild deficit group demonstrated significantly higher total SINS scores (P = 0.024) and a greater prevalence of intermediate and unstable SINS (P = 0.049). In contrast, ESCC grade distribution did not show a statistically significant difference.

**Table 4:** Comparison of characteristics between the patient groups with various severities of spinal cord injuries.

	Mild deficit (n = 22)	Moderate to severe deficit (n = 18)	P-value
Clinical			
Pain character, n (%)			
Localized pain	8 (36.4)	8 (44.4)	0.604
Mechanical pain	9 (40.9)	4 (22.2)	0.209
Radicular pain	12 (54.5)	7 (38.9)	0.324
Period to onset, months, mean (SD)	4.5 (3.8)	4.5 (3.7)	0.840
Radiologic			
SINS, total score, mean (SD)	9.5 (3.8)	6.9 (2.9)	0.024
SINS, category, n (%)			
Stable	6 (27.3)	10 (55.6)	
Intermediate	11 (50.0)	8 (44.4)	
Unstable	5 (22.7)	0 (0.0)	0.049
ESCC scale, n (%)			
0	4 (18.2)	6 (33.3)	
1	5 (22.7)	7 (38.9)	
2	7 (31.8)	3 (16.7)	
3	6 (27.3)	2 (11.1)	0.306

The Student’s *t*-test, Mann–Whitney U test, Pearson’s χ2 test, and Fisher’s exact test were used to compare variables between the patient groups.

SD, standard deviation; SINS, spinal instability neoplastic score; ESCC, epidural spinal cord compression.

### 3.4 Period to the onset of spinal cord injuries

The interval from MRI to the onset of spinal cord injury was analyzed in the spinal cord injury group (n = 40) based on the SINS and ESCC grades. The results are presented in **Table 5** as secondary outcomes. When categorized by the SINS, no significant ordered differences in the period were observed (P = 0.575). However, when categorized by ESCC grade, the average period to spinal cord injury onset decreased with increments in grades (grade 0: 6.8 months; grade 1: 4.7 months; and grade 2 or 3 [high-grade]: 3.1 months). This trend was statistically significant (P = 0.003).

**Table 5:** Comparison of the period to the onset of spinal cord injuries based on different SINS and ESCC scales.

	Period, months, mean (SD)	P-value
SINS, category		
Stable	4.6 (3.8)	
Intermediate	4.3 (3.5)	
Unstable	4.8 (5.2)	0.575
ESCC scale		
0	6.8 (4.1)	
1	4.7 (3.4)	
2 or 3 (High-grade)	3.1 (3.2)	0.003

The Jonckheere–Terpstra test was used to analyze the ordered difference in the period to neurological complication onset in each patient group with different SINS and ESCC scales.

SD, standard deviation; SINS, spinal instability neoplastic score; ESCC, epidural spinal cord compression.

## 4 Discussion

To our knowledge, this is the first study to investigate predictive factors for the occurrence of spinal cord injury within 1 year in patients with metastatic spinal tumors. We analyzed clinical factors related to pain, as well as radiologic data derived from the SINS and ESCC scale, alongside the timing and severity of spinal cord injury, to identify associated factors for each. Unlike previous studies that primarily analyzed spinal cord injuries in patients with metastatic spinal tumors after occurrence, this study focused on identifying predictive factors for new spinal cord injuries within 1 year (14–17). In clinical practice, patients with spinal metastases often undergo clinical management and whole-spine MRI before the manifestation of spinal cord injury. Therefore, our findings provide valuable insights and clinical implications for managing spinal metastases.

Our institution, a tertiary hospital in Seoul, Korea, operates a multidisciplinary Bone Metastasis Clinic dedicated to the comprehensive management of patients with bone metastasis (7). According to our institution’s clinical guidelines, whole-spine MRI is recommended for further evaluation when spinal metastasis is detected during the evaluation of a primary cancer or when patients present with vertebral pain or clinical suspicion of neurological symptoms.

Among the study population, over 70% of the primary cancers were breast, lung, prostate, kidney, and liver cancers— the malignancies most commonly associated with bone metastasis at advanced stages, particularly breast, lung, prostate, and kidney cancers (7, 14, 23, 24). Additionally, bone metastasis predominantly involved the thoracic spine, consistent with previous literature (7, 14, 17, 24).

Radicular pain was observed in 47.5% of patients who developed spinal cord injury, compared to 20.0% of patients who did not. The multivariate analysis identified radicular pain as a marginally significant predictor of spinal cord injury (P = 0.053). Radicular pain likely indicates nerve root impingement caused by the metastatic spinal tumor or pathologic vertebral fractures, as well as an elevated risk of adjacent spinal cord compression and injury (18). A previous study also identified radicular pain as the most common initial symptom of metastatic spinal cord compression (25). Guidelines for spinal metastasis recommend immediate spinal MRI for patients with bilateral radicular pain due to the possibility of spinal cord compression (20). Thus, patients with spinal metastases and radicular pain may benefit from shorter clinical evaluations, timely preventive surgery, and comprehensive patient education regarding alarm symptoms.

The radiologic indices, the SINS and ESCC grades, are well-established tools for assessing spinal instability and the mass effect of tumors in patients with spinal metastasis (24). Both indices have demonstrated high intraobserver and interobserver reliability (8, 13, 24, 26, 27). Pathologic vertebral fractures and direct tumor invasion are the primary causes of spinal cord compression and injury. This study evaluated the predictive value of the SINS and ESCC grades in identifying this injury process.

The association between the SINS and neurological outcomes or prognosis has been inconsistently reported in previous literature (14, 15, 17). We observed a significant difference in the distribution of SINS categories between patients with and without spinal cord injuries. Specifically, all five patients with unstable SINS developed spinal cord injuries within 1 year post-MRI. However, when analyzing injury severity, a higher SINS category was significantly associated with milder neurological deficits. This may be attributed to the retrospective nature of the study, in which treatment of metastatic spinal tumors was uncontrolled. Four of five patients with unstable SINS underwent early surgical intervention for severe pain and spinal instability. These findings suggest that, while earlier surgical intervention for unstable spines, as recommended by current clinical guidelines, may not entirely prevent spinal cord injury within 1 year post-MRI, it could help mitigate the severity of neurological deficits (6, 9, 20).

Spinal cord injury was significantly more prevalent among patients with higher ESCC grades. Notably, high-grade ESCC was identified as the only significant predictor of spinal cord injury within 1 year in the multivariate analysis. Additionally, the interval between MRI and spinal cord injury onset decreased with increasing ESCC grade. Given that the ESCC scale assesses the degree of spinal cord compression, these findings are readily explainable. However, no statistically significant association was observed between ESCC grade and spinal cord injury severity. These findings are consistent with those of a previous study on spinal metastases, which reported that ESCC grade was not associated with the degree of paralysis, whereas high-grade ESCC was identified as a risk factor for rapidly progressive paralysis (16).

A notable finding was that spinal cord injury occurred within 1 year in 16 (69.6%) of 23 patients with stable spines and 22 (45.8%) of 48 patients with ESCC grade 0 or 1; over half of these patients exhibited moderate to severe deficits. Current guidelines recommend systemic therapy and radiation therapy over surgery for early lesions of spinal metastases, suggesting less intensive treatment and follow-up protocols compared to those for patients with advanced lesions (8, 20, 28). However, our study findings suggest a need for a more active therapeutic approach, even for patients with early lesions, to prevent moderate to severe neurological deficits. For instance, the average time to spinal cord injury was 4.6 months for patients with stable SINS and 6.8 months for those with ESCC grade 0, suggesting that an earlier routine imaging follow-up protocol could be beneficial. Further research is needed to investigate the cost-effectiveness of additional imaging studies and more proactive interventions for early spinal metastases.

This study has some limitations. First, treatment for spinal metastases was uncontrolled among patients with and without spinal cord injury. Patients in the injury group, who were classified under the higher SINS categories and ESCC grades, more frequently underwent surgery. This limitation stems from the retrospective design of the study and may have confounded the results. Further controlled and prospective studies would be helpful to validate the results of this study. Second, selection bias may have occurred, as only patients referred to the Department of Rehabilitation Medicine were included. These individuals may have had a worse prognosis or more advanced cancer compared to the general population of patients with spinal metastasis. Indeed, the proportion of patients in this study who developed spinal cord injury (40 of 70 patients, 57.1%) was much higher than the previously reported rate of approximately 12%–16% (1, 29). Third, the relatively small sample size limited the statistical power of the multivariate analysis. To address this limitation, possible predictors based on a univariate analysis threshold of P < 0.05 were included, followed by stepwise variable selection to reduce the risk of overfitting. The study was designed with reference to previous studies with comparable sample sizes, event numbers, and numbers of variables included in multivariate analyses (12, 14). Fourth, the clinical context in which whole-spine MRI was performed varied among included patients. At our institution, whole-spine MRI is typically recommended for further evaluation when spinal metastasis is detected during the primary cancer workup or when vertebral pain or suspected neurological symptoms are present. Although this may have confounded our study, it reflects real-world clinical practice. Further multi-center studies would be helpful to validate the generalizability of our findings. Fifth, retrospective pain data collection may have been inaccurate. Finally, for patients who underwent radiation therapy, the impact of radiation-induced myelopathy cannot be excluded. However, as this condition is typically delayed in onset and cumulative in nature, its impact is likely minimal (30).

## 5 Conclusion

High-grade ESCC was identified as a significant predictor of spinal cord injury within 1 year and was associated with an earlier onset of injury. Radicular pain and unstable spines based on SINS categories were also strongly associated with spinal cord injury. This study provides valuable insights for predicting 1-year functional outcomes and guiding management strategies for spinal metastases.

## Data Availability

The raw data supporting the conclusions of this article will be made available by the authors, without undue reservation.
