# Supplementary material for: Can we predict neurological complications in patients with metastatic spinal tumors?
Source: Front Oncol. 2025 Aug 19;15:1625545. doi: 10.3389/fonc.2025.1625545 (PMC12402062; doi:10.3389/fonc.2025.1625545)
Supplement: Supplementary file 1 [file Table1.docx]

Supplementary Material

# Supplementary Table 1. Spinal instability neoplastic score (SINS)

| Component of SINS | Score |
| --- | --- |
| Location |  |
| Junctional (occiput-C2, C7-T2, T11-L1, L5-S1) | 3 |
| Mobile (C3-C6, L2-L4) | 2 |
| Semi-rigid (T3-T10) | 1 |
| Rigid (S2-S5) | 0 |
| Pain |  |
| Mechanical | 3 |
| Occasional and nonmechanical | 1 |
| Pain-free | 0 |
| Bone lesion type |  |
| Lytic | 2 |
| Mixed (lytic/blastic) | 1 |
| Blastic | 0 |
| Radiographic spinal alignment |  |
| Subluxation/translation | 4 |
| Kyphosis/lordosis | 2 |
| Normal | 0 |
| Vertebral body collapse |  |
| >50% collapse | 3 |
| <50% collapse | 2 |
| No collapse with >50% body involvement | 1 |
| None | 0 |
| Posterolateral involvement of spinal elements |  |
| Bilateral | 3 |
| Unilateral | 1 |
| None | 0 |
| Total | 18 |

* Fisher CG, DiPaola CP, Ryken TC, Bilsky MH, Shaffrey CI, Berven SH, Harrop JS, Fehlings MG, Boriani S, Chou D, Schmidt MH, Polly DW, Biagini R, Burch S, Dekutoski MB, Ganju A, Gerszten PC, Gokaslan ZL, Groff MW, Liebsch NJ, Mendel E, Okuno SH, Patel S, Rhines LD, Rose PS, Sciubba DM, Sundaresan N, Tomita K, Varga PP, Vialle LR, Vrionis FD, Yamada Y, Fourney DR (2010) A novel classification system for spinal instability in neoplastic disease: an evidence-based approach and expert consensus from the Spine Oncology Study Group. Spine (Phila Pa 1976) 35:E1221-E1229.

# Supplementary Table 2. Epidural Spinal Cord Compression (ESCC) scale

| ESCC scale | Description |
| --- | --- |
| ESCC grade 0 | Bone-only disease |
| ESCC grade 1a-c | Impingement or deformation of the dural sac, without spinal cord compression |
| ESCC grade 2 | Spinal cord compression, but with CSF visible around the cord |
| ESCC grade 3 | Spinal cord compression, no CSF visible around the cord |

* Bilsky MH, Laufer I, Fourney DR, Groff M, Schmidt MH, Varga PP, Vrionis FD, Yamada Y, Gerszten PC, Kuklo TR: Reliability analysis of the epidural spinal cord compression scale. Journal of Neurosurgery: Spine 2010, 13(3):324-328.
